# Topological analysis of interaction patterns in cancer-specific gene regulatory network: persistent homology approach

**DOI:** 10.1038/s41598-021-94847-5

**Published:** 2021-08-12

**Authors:** Hosein Masoomy, Behrouz Askari, Samin Tajik, Abbas K. Rizi, G. Reza Jafari

**Affiliations:** 1 Physics Department, Shahid Beheshti University, Tehran, Iran; 2 Physics Department, Brock University, St. Catharines, ON L2S 3A1 Canada; 3 Department of Computer Science, School of Science, Aalto University, 0007 Espoo, Finland; 4 Department of Network and Data Science, Central European University, Budapest, 1051 Hungary

**Keywords:** Biological physics, Complex networks

## Abstract

In this study, we investigated cancer cellular networks in the context of gene interactions and their associated patterns in order to recognize the structural features underlying this disease. We aim to propose that the quest of understanding cancer takes us beyond pairwise interactions between genes to a higher-order construction. We characterize the most prominent network deviations in the gene interaction patterns between cancer and normal samples that contribute to the complexity of this disease. What we hope is that through understanding these interaction patterns we will notice a deeper structure in the cancer network. This study uncovers the significant deviations that topological features in cancerous cells show from the healthy one, where the last stage of filtration confirms the importance of one-dimensional holes (topological loops) in cancerous cells and two-dimensional holes (topological voids) in healthy cells. In the small threshold region, the drop in the number of connected components of the cancer network, along with the rise in the number of loops and voids, all occurring at some smaller weight values compared to the normal case, reveals the cancerous network tendency to certain pathways.

## Introduction

Cancer is one of the most common human genetic diseases characterized by cellular over-proliferation^1–3^. Through the gene expression process, genetic code modulates biological functions and associated molecular pathways. The subsequent cellular phenotype is modulated by a dynamic network of interactions among genes. Perturbations in these interactions affect the overall manifestation of genetically driven diseases such as cancer. Genes and their dynamic interactions can be modeled by complex networks represented by nodes and links^4^. In network systems, each node is considered as a dynamic entity, evolving under the influence of others^5–9^. Systems of interacting units consist of links having positive, negative, or zero weight and they together develop a weighted signed network, called Gene Regulatory Network (GRN)^10–14^. GRNs can be constructed by maximum entropy models, analyzed by balance theory approaches^15^ and topological methods^16^. Moreover, responses to driving forces on the structure formation of these networks cause the development of new features and subsequently lead to the identification of unique patterns in the observational data. These patterns can arise from non-trivial connections that go beyond classical pairwise interactions, leading to a higher-order construction^16^. These constructions can be described by simplices of different dimensions and hence, can be studied in the framework of Balance Theory and Topological Data Analysis (TDA). From TDA, we employ the Persistent Homology (PH) analysis tool, which is based on algebraic topology and has been applied to problems in a variety of fields such as network science, physics, chemistry, biology, and medicine^17–31^. PH has been previously used to study protein-protein interaction networks to inform cancer therapy by determining the correlation between Betti numbers and the survival of cancer patients^32^.

In order to study states of balanced and imbalanced cancer networks, we previously modeled GRNs by groups of three interacting genes, forming triangles (triads) of interactions^15^. The resulting signed weighted network analysis in the context of Balance Theory showed significant differences between cancer and healthy cases of GRNs in the number of characteristics such as energy, number, and distribution of imbalanced triangles. This paper aims to study the higher-order representation of gene regularity interaction networks derived from cancer and normal samples. Using PH, we address theoretical concepts using empirical data and report network features of cancer samples compared to normal counterparts. Finally, we propose PH as unsupervised network analysis to study human diseases such as cancer.

## Network construction from real data and the result of balance theory analysis of the interaction network

Gene expression is the process by which information from a gene is used in the synthesis of a functional gene product which leads to the production of protein as the final functional product. Cells go through a wide range of mechanisms known as Gene regulation to increase or decrease the production of specific gene products. Gene expression data is large-scale measurements of the degrees of freedom of a biological system such as a cell. In the language of statistical physics, these describe the micro-states of a cell. A gene regulatory network is a complex network^33^ which its nodes represent the genes, and its links between them represent the interactive couplings between genes which can be used to predict the global properties of the cells.

We used mRNA (expression level) data of 20532 genes in the case of Breast Cancer (BRCA: Breast invasive carcinoma) from The Cancer Genome Atlas (TCGA)^34,35^. Since RPKM (Reads Per Kilobase transcript per Million reads) puts together the ideas of normalizing by sample and by gene, we used the RPKM normalized data to find the correlation between the expression levels of the genes. The Reads Per Kilobase transcript per Million reads (RPKM) normalized data was used in order to put together the ideas of normalizing by sample and by the gene. When we calculate RPKM, we are normalizing for both the library size (the sum of each column) and the gene length. Due to computational purposes, we only kept the top 483 most variable genes for all analyses by calculating for each gene the variance of its expression level over its samples. For each gene, we have calculated the variance of its expression level over its samples, and accordingly stored the first 483 genes with the highest variance, which is due to more different activity patterns these genes show. This cohort consisted of two sets of 114 healthy and 764 cancer samples.

We constructed a pairwise correlation matrix^36,37^ from our data-set based on pairwise gene expressions in the obtained data-set. To find the regulatory connections between genes, we needed a statistical description of the data in terms of suitable observables and infer^38,39^ the underlying regulatory connections. Therefore, we restricted ourselves to an undirected pairwise maximum-entropy probability model with terms up to second order^40–42^, which we derive for continuous, real-valued variables. This can be considered as a problem in inverse Statistical Physics^43,44^ where we want to infer parameters of a model based on observations, instead of calculating observables on the basis of model parameters. We applied the following model with pairwise couplings

1 $$\begin{aligned} P(\{S_i\}) = \frac{1}{Z} \exp {\sum _{i<j}{J_{ij}~S_i~S_j}} \end{aligned}$$

where $S_i$ represents the expression level of gene *i* as a continuous real-valued variable, and interaction matrix $J_{ij}$, describes the strength of the net interaction between two genes. *Z* is the so-called partition function, for normalizing the model. The corresponding Hamiltonian (energy function) for this Boltzmann distribution function is then $H = -\sum _{i<j}{J_{ij}~S_i~S_j}$.

Model parameters can be found by satisfying these conditions through the use of Lagrange multipliers; (i) Our model should give the same first and second moments as we measure from the data and (ii) it must maximize the Gibbs-Shannon entropy function defined as $S[P] = -\sum P(\{S_i\})\ln (P(\{S_i\}))$. The obtained model is a multivariate Gaussian distribution of the form:

2 $$\begin{aligned} P(S;\langle S \rangle ,C)= \frac{\exp \left[ {-\frac{1}{2}(S-\langle S \rangle )^TC^{-1}(S-\langle S \rangle )} \right] }{{(2\pi )^{L/2} \ {\det (C)}}^{1/2}}, \end{aligned}$$

where *L* is the number of genes in the distribution and the couplings can be inferred simply by inverting the matrix of variances and covariances of expression levels $J_{ij}= - {C^{-1}_{ij}}$. This approach is also linked to the concept of partial correlations in statistics^45,46^ such that the inverse of the covariance matrix, $C^{-1}$, also known as precision matrix, offers information about the partial correlations of variables.

By assuming a maximum entropy pairwise model, we were looking for the interaction matrix J, whose every element $J_{ij}$ is the strength of the net interaction between gene i and gene j. In other words, the strength and the sign of the interaction represents the mutual influence of a pair of genes’ expression levels on one another. In real data samples, we considered genes that are either expressed or not expressed together, and defined them as being correlated when they are expressed (or not expressed) mutually. Subsequently, one can construct correlation matrices. However, concerning the interaction matrix construction, we need a model Hamiltonian, producing coefficients. Hence, from the experimental data, we reconstruct the gene-gene interactions computationally based on a model, following the practice that collective behaviors in such systems are described quantitatively by models that capture the observed pairwise correlations. Elements of the proposed interaction matrix *J*, represent pairwise interaction between genes in the proposed model, where the weight of the link $i - j$, represented by $J_{ij}$ denote the strength of the interaction between gene *i* and gene *j*. Furthermore, genetic interaction (GI) between two genes can be inferred from the sign of their interactions, indicating the way they may affect each other’s functions. Positive and negative interactions on the foundation of the constructed network imply gene expression modulation within the network. Therefore, we expect *J* to be a sparse matrix since each gene interacts only with a couple of other genes. Inverting a large covariance matrix computationally, however, yields to a matrix which almost none of its elements are zero. To keep this at bay, the inverse of the covariance matrix has been obtained by means of the Graphical Lasso (GLasso) algorithm^47^. GLasso is generally a sparse penalized maximum likelihood estimator for the concentration or inverse of covariance matrix of a multivariate elliptical distribution. When dealing with a multivariate Gaussian distribution with limited observations (lack of enough samples)^48,49^, GLasso yields a sparse network ($-C^{-1}$) while preserving the global features of the network^50^. In a network analysis, simple thresholding methods can be misleading because removing weak ties may results in the fragmentation of the network; A pair of genes may be weakly connected, while that tie plays a significant role in the structure of the network. On the other hand, removing a strong connection between insignificant or isolated pair of nodes may not destroy the global features of the network, G-Lasso is wary of such issues.

According to structural balance theory, dyadic links holding positive and negative interactions yields four different types of triads, triangles of interactions, in the network^51–55^. Balance and imbalanced states of triangles are consequently determined based on the sign of the product of the links; balanced when positive ($J_{ij}J_{jk}J_{ki} > 0$), and imbalanced or frustrated otherwise ($J_{ij}J_{jk}J_{ki} < 0$), and their corresponding energy of a triangle, being defined as $E_{ijk} = J_{ij}J_{jk}J_{ki}$, constructs an “Energy landscape” for the network. The stability of imbalanced triangles in the GRN has been studied in previous reports and complex structures and collective behavior of genes has been examined. Previous results confirmed that cancerous cells posses a fewer number of imbalanced triangles compared to the normal samples. In addition, imbalanced triangles in the healthy network appear to be more isolated from the main part of the network. It was shown how the distributions of triangles in the network and their absolute corresponding energy can be used as means to compare normal and cancer networks^15^.

Stability, in terms of Balance Theory corresponds to a lower energy level according to the proposed energy equation^56–59^. It implies less possibility of changing the configuration of the triangles and therefore, less change in gene regulation within the network. The energy landscape of networks was previously proposed to examine the state of balance^60^. Energy distributions of different types of triangles was significantly variable in cancer samples compared to normal counterparts. In addition, it was found that the cancer network has less tendency to change its state due to its lower energy level compared to normal network^15^.

Examining the distribution of the triangles suggested the correlations between such triangles were also different between the two networks^15^. Based on this observation, we asked how triads with different energies are connected to one another and how schematic diagrams of distribution of frustrated triangles in the normal and cancer network differ. To address this, the concept of exceeding the length of interaction from triplet interactions towards higher-order interactions, quartic interactions or Energy-Energy Correlation between triangles can be proposed, allowing one to study the very influence of units of four entities on the final degree of balance^61^. Considering a simple pairwise interaction term between triads with a common edge in previous reports, the model Hamiltonian to treat the states of balanced and imbalanced triads is defined

3 $$\begin{aligned} H= - \sum _{i<j<k<l}\Delta _{ijk}~\Delta _{ijl}=-s(G), \end{aligned}$$

where $\Delta _{ijk}$ represents a triad shaped by *i*, *j*, *k* nodes. In quartic balance theory now the number of squares, i.e., *s*(*G*), is an essential parameter for the specific graph configuration and according to structural balance, the corresponding energies can be compared. This formalism examines the probability distribution of the jammed states’ levels of energy, assuming that for the triads, the shift from balanced to imbalanced can be determined based on all triads that share a common link^61^. As discussed, constructing 3rd and 4th order interaction networks and examining their corresponding energy for normal and cancer networks provide us with practical insights and can be used to compare stability, energy, and the tendency toward changing their states in cancer and normal samples. This concept motivated us to move forward to study higher-order interaction methods, so as to gain a thorough perspective of these interactions, the patterns of these interactions, by which we address further unsolved questions in this matter. In this paper, as an alternative to studying higher dimensional simplices, we employed a topological scheme to examine the interaction patterns of two networks. This method involves studying cancer and normal gene networks using behaviours of defined *k*-dimensional holes as a general approach to study their higher-order interactions.

## Method

**Figure 1:**
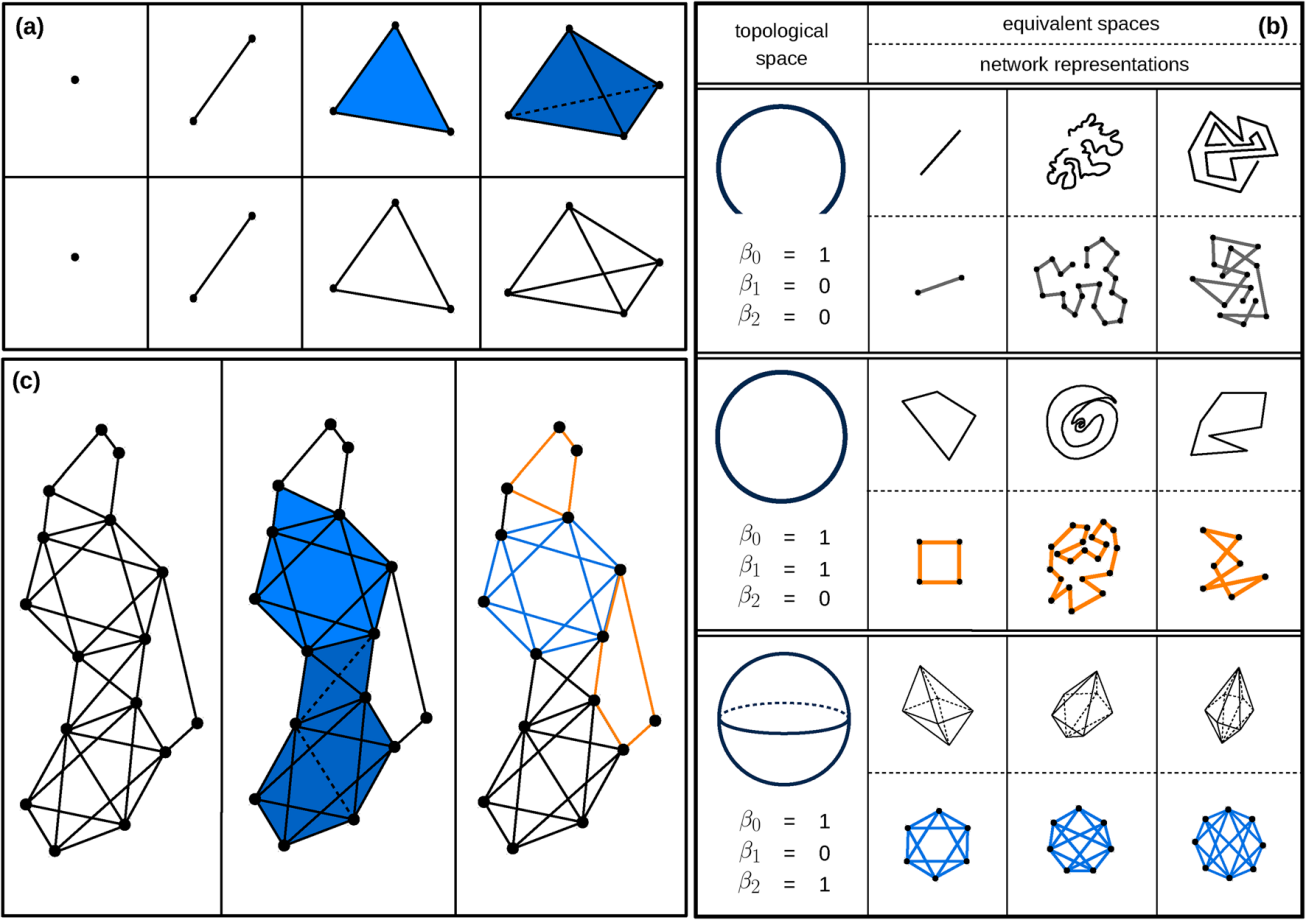
**(a)** 0-,1-,2-, and 3-simplex from left to right (Up row), and their network representation (bottom row). **(b)** Example of some topological spaces with their associated Betti numbers (left column), and the equivalent spaces and their network representation (right column). **(c)** An example for constructing the associated clique simplicial complex (middle column) from an unweighted network of nodes and links (left column), and its network representation with its topological features(right column). In network representation, orange and blue subnetworks correspond to 1-holes (loops) and 2-holes (voids), respectively. The network has the Betti vector of $\vec \beta = (1,2,1)$.

By analogy, studying and comparing patterns of interactions in the networks as an alternative to transcending triads or quartic order can be considered as describing a building by its floors and bedrooms, and hallways rather than its building blocks. To study higher-order interactions in cancer networks, formed not only by nodes and links but also by triangles and cliques of higher dimensions, we employed algebraic topology strategy toward analyses that require the encapsulation of higher dimensionality as a substitute for simple pairwise interactions. We suggest that these representations are implemented to complement our previously employed network techniques to distinguish the features of cancerous and normal networks. Here we preview some fundamentals of algebraic topology, and homology theory that is utilized in topological data analysis^62–65^. A simplicial complex is represented by a set of a finite collection of *k*-dimensional simplices (*k*-simplices) $\sigma _{k}=[v_0,v_1,\ldots ,v_k]$. In Fig. 1 we show the configuration of low-dimensional simplices, their network representation, constructing the associated clique simplicial complex from an unweighted network of nodes and links, and their topological features. As it can be noted from the figure, a 0-simplex $\sigma _{0}$ is regarded as vertex (node), a 1-simplex $\sigma _{1}$ is defined as an edge (link), a 2-simplex $\sigma _{2}$ is a triangle, and a 3-simplex $\sigma _{3}$ is a tetrahedron, and so on, see Fig. 1a. For a given simplicial complex $\psi$, one can define a *k*-dimensional chain (*k*-chain) as a linear combination of *k*-simplices of $\psi$ as follows:

4 $$\begin{aligned} c_{k} = \sum _{i} a_i \sigma _{k}^{(i)}, \end{aligned}$$

where the coefficient $a_i \in {\mathbb {Z}}_2$ and the sum is over all *k*-simplices $\sigma _{k}$ in $\psi$. It can be considered that a set of *k*-simplices forms an abstract vector space $C_k$, so-called *k*-dimensional chain group (*k*-chain group), where its dimension is the number of *k*-simplices of the complex. For any simplices in any dimension *k*, in order to measure the topological features and study the homology of the complex a *k*-dimensional boundary operator has to be defined as:

5 $$\begin{aligned} \partial _k (\sigma _k) = \sum _{i=0}^{k} (-1)^i~[v_0 ,\ldots , v_{i-1} , v_{i+1} ,\ldots , v_k] ~ \subseteq ~ \sigma _{k} \end{aligned}$$

So $\partial _k$ is an operator, mapping $\sigma _k$ to its boundary and consequently *k*-dimensional chain group $C_k$ to $(k-1)$-dimensional chain group $C_{k-1}$:

$$\begin{aligned} ... ~ \xrightarrow {\partial _{k+2}} C_{k+1} \xrightarrow {\partial _{k+1}} C_{k} \xrightarrow {\partial _{k}} C_{k-1} \xrightarrow {\partial _{k-1}} ~ ... \xrightarrow {} C_{2} \xrightarrow {\partial _{2}} C_{1} \xrightarrow {\partial _{1}} C_{0} \xrightarrow {\partial _{0}} \emptyset \end{aligned}$$

One can define a *k*-dimensional cycle (*k*-cycle) $z_k$ as a *k*-chain $c_k$ that is mapped to empty set by boundary operator, $\partial _k(c_k)=\emptyset$. This leads to create a subspace $Z_k$, so-called *k*-dimensional cycle group (*k*-cycle group), of vector space $C_k$. On the other hand a *k*-chain $c_k$ that is the boundary of a $(k+1)$-chain $c_{k+1}$ can be define as a *k*-dimensional boundary (*k*-boundary) $b_k$ and consequently *k*-dimensional boundary group (*k*-boundary group) $B_k$ as subspace of $C_k$. Since “boundaries have no boundary”, one can easily write $B_k \subseteq Z_k \subseteq C_k$. The idea of homology theory is to discard *k*-cycles that are also *k*-boundary. To this end, we put an equivalence relation on $Z_k$ as follows. Two *k*-cycles $z_{k}^{(i)}$ and $z_{k}^{(j)}$ are homologous (equivalent), $z_{k}^{(i)} \sim z_{k}^{(j)}$, if $z_{k}^{(i)} - z_{k}^{(j)} \in B_k$. The equivalence relation $\sim$ partitions the subspace $Z_k$ into a union of disjoint subsets, called homology classes. The *k*-homology group of complex $\psi$ is defined as $H_k \equiv \{[z_k] ~ | ~ z_k \in Z_k\}$ where $[z_k]$ is the homology class of $z_k \in Z_k$.

6 $$\begin{aligned} H_k = Z_k / B_k \end{aligned}$$

**Figure 2:**
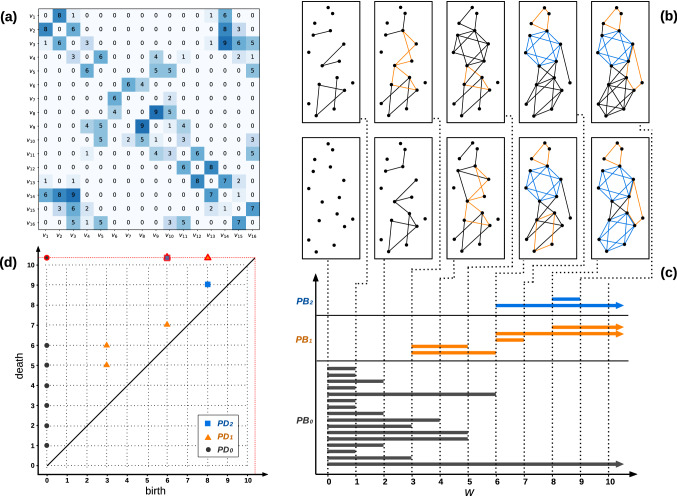
**(a)** An example of the adjacency matrix for a weighted network. The shade of each pixel corresponds to the weight of the link between the associated nodes. **(b)** A filtration for the weighted clique simplicial complex constructed from the weighted network. **(c)** Persistence barcode representing the topological evolution of *k*-dimensional topological holes. Evolution (birth-weight and death-weight) of any *k*-holes in the filtration are represented by a horizontal bar ($k=0,1,2$ black, orange and blue bar, respectively), starting from its birth-weight and ending at its death-weight. The arrows indicate the survived holes. **(d)** Persistence diagram for the filtration. Any *k*-hole in the filtration is shown by a point ($k=0,1,2$ black circle, orange triangle, and blue square, respectively), called persistence pair, in 2-dimensional Euclidean space, known as birth-death space. The first and the second element of the persistence pair equals birth-weight and death weight, respectively. The survived holes lie on the horizontal red dashed-line.

The *k*th Betti number of complex $\psi$, denoted by $\beta _{k}(\psi )$, as a topological invariant of the complex, is the dimension of *k*-homology group of the complex $\psi$. Intuitively $\beta _{k}(\psi )$ indicates number of *k*-dimensional topological holes (*k*-holes) of complex $\psi$. Thus $\beta _{0}$ counts number of connected components of $\psi$, $\beta _{1}$ counts number of 1-holes (loops) of $\psi$, $\beta _{2}$ counts number of 2-holes (voids) of $\psi$ and so on, see Fig. 1b. In the graph representation of Fig.1, the one-dimensional topological hole and the two-dimensional hole are illustrated with orange and blue colored lines respectively as our starting point to schematically define these topological features and identify them. It then follows that the Betti numbers are used as an algebraic tool in order to classify the topological spaces and study the homology of the complex^66^. Due to studying complex networks in terms of homology theory, we use the persistent homology (PH) technique which is the main part of topological data analysis (TDA) as a modern mathematical tool in data science. Following persistent homology strategy, rather than working with the set of nodes (1-simplices) and links (2-simplices), and the statistical properties of the network defined in network science^67^, we consider higher-order connections as high-dimensional simplices to map the network. In fact, a clique simplicial complex of a network is a simplicial complex in which any *k*-simplex $\sigma _{k}$ corresponds to a $(k+1)$-clique (a complete sub-network of order $k+1$), Fig. 1c. In order to analyze the impact of weight in the structure of a complex network, PH considers the weight as the filtering parameter (threshold), so the filtration as an increasing sequence of complexes can be created, such that, all 1-simplexes (links) with weights higher than the threshold are removed from the weighted complex (network). Upon this development, various topological features such as 1-dimensional holes (loops), and 2-dimensional holes(voids) will appear (birth) by changing the threshold, where they may later disappear (death) in higher values. During a filtration, by varying the threshold of interaction *w*, a topological feature $h_k$ may appear $w_{b}^{(h_k)}$, or disappear $w_{d}^{(h_k)}$; and the persistency (lifetime) $l^{(h_k)} \equiv w_{d}^{(h_k)} - w_{b}^{(h_k)}$ of these homological features can be used to analyze global features of the data-set, which in our case is to examine the differences between the two data-sets^29,68^. Persistence barcode (PB) or equivalently persistence diagram (PD) for each dimension, are representations of PH that summarize topological information of the data-set. For instance, in PD plot of *k*th dimension for weighted complex $\psi (w)$, any topological feature $h_{k}$ is represented by a point $p^{(h_{k})}=(w_{b}^{(h_k)},w_{d}^{(h_k)})$, persistence pair, in a 2-dimensional Euclidean space. Figure 2 elaborates the filtration process and the evolution of *k*-dimensional topological holes and their persistence upon increasing the threshold by the mean of persistence diagram and barcode for the filtration. By this approach, one can capture global features of the network at any threshold (weight) and monitor the persistence and the robustness of the topological features. Hence, adopting a simplicial modeling, a gene is defined as a 0-simplex $\sigma _{0}$, and the interactions between genes are regarded as a 1-simplex $\sigma _{1}$, and so on. Through varying the scale over which the connections between vertices are made, we aim to identify the behavior of defined simplices from one another within two networks. In a network of interaction, where the genes are vertices and the interactions between two genes are defined as edges, we impose PH to map the network to a weighted clique simplicial complex, use the strength of interaction as a varying threshold, and obtain a family of complexes (subcomplexes) as a function of the weight. We establish a family of unweighted graphs where their topological features can be examined, and their topological evolution as a function of interaction threshold can be studied. This approach can be taken as an alternative to assigning a Hamiltonian to a weighted interaction network to compare these two networks topologically rather than quantitatively in terms of their energy landscape.

## Result and discussion

**Figure 3:**
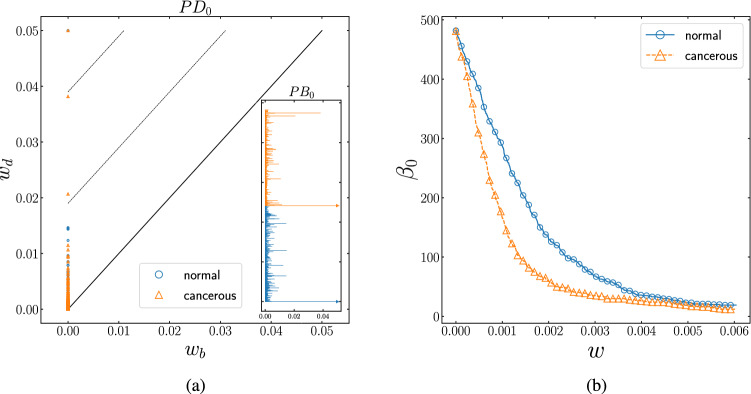
**(a)** Persistence diagram of 0-homology group ($PD_{0}$) for the normal (blue circles) and the cancer (orange triangles) gene interaction network. The cancerous network includes two small persistent clusters (orange triangles between dashed-lines). Inset: Corresponding persistence barcode $(PB_{0})$ for normal (blue bars) and cancerous (orange bars) network. The number of survived connected component (arrows) indicate that both networks are path-connected, and two orange long bars correspond to the small persistent clusters in cancerous network. **(b)** The number of connected components as a function of threshold ($\beta _{0}$-curve) for the normal (blue circle) and the cancer (orange triangle) network. This curves indicate that the cancer network has more global accessibility rather than the normal network. The number of connected components in the cancer data-set dropped at a smaller value.

By analyzing the interaction networks from the topological point of view, we aim to uncover prominent insights into cellular gene interaction patterns. To this end, applying the PH technique on the weighted complex networks of the normal and cancerous data sets, we analyze the evolution of the dimension of the *k*-homology group of the topological space ($\beta _{k}$); where these Betti numbers demonstrate the number of *k*-dimensional topological holes. As previously noted, a *k*-hole of the space, depending on its dimension, is a subspace that has no boundary and is not a boundary of any spaces. From the complex network perspective, the *k*-holes indicate a lack of higher-order connections (links, triangles, ...) between the nodes (agents) of the network, such that by increasing the number of 0-holes $\beta _{0}$ (connected components), one can discuss about the lack of links (1-simplices) to connect the connected components. Whereas, arising the number of 1-holes $\beta _{1}$ (topological loops) implies the lack of triangles (2-simplices) to connect the nodes (agents) of a sub-network. Through extracting the homological features as a set of evolving $0-2$ dimensional Betti numbers, we compare two gene regulatory networks’ interaction patterns topologically. Measuring the number of independent holes of dimension *k*, plotting their persistence barcode and persistence diagrams and their evolution as a function of weight, is our key point to analyze the topological features of these two data sets. Figure 3 shows the evolution of the number of connected components (0-dimensional holes), its topological barcode, and the persistent diagram for both networks as a function of threshold. As the absolute value of the threshold was increased from 0, there was a sudden decrease in the number of components for both networks. For the cancer network, this sudden drop appeared to happen in a smaller value of interaction.

**Figure 4:**
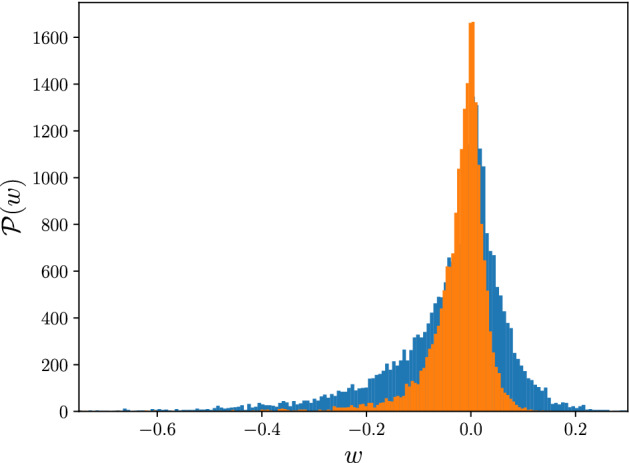
Distribution of weights of links for both normal (blue) and cancerous (orange) networks, where the shorter width of the distribution function of cancerous sample compared to the normal one indicates that the normal network has high-weighted links (tail of the distribution functions) rather than the cancerous network.

**Figure 5:**
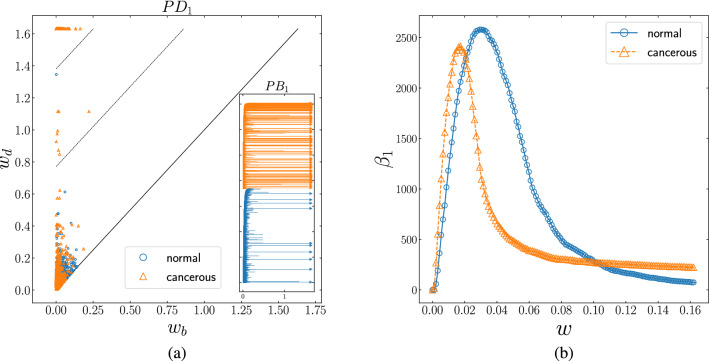
**(a)** Persistence diagram of 1-homology group ($PD_{1}$) for normal (blue circles) and cancerous (orange triangles) interaction network. There are many persistent loops (persistence pairs between dashed-lines) in cancerous network rather than normal network. Inset: Corresponding persistence barcode ($PB_{1}$) for normal (blue bars) and cancerous (orange bars) network. The long bars correspond to the persistent loops in normal and cancerous networks, and the number of survived loops (arrows) in the cancerous network is more than the normal network. **(b)** The number of topological loops as function of threshold ($\beta _{1}$-curve) for normal (blue circle) and cancerous (orange triangle) network. The networks become loop-full at different thresholds (0.02 and 0.03 respectively), whereas they include the same number of loops almost at 0.02 and 0.10. More importantly, the tail of curves show that the cancerous network is loopful, but the normal network is almost loopless.

We then asked whether this apparent separation was due to the variation in the strength and distribution of links in those two networks, where the range of weight function seems to be shortened in the cancerous data and more scattered in the normal one, Fig. 4. We found that the faster decline of established components of gene expression interactions in the cancer network is driven by links with the smaller weight. It is noted that gene interactions with higher weight values play a crucial role in the normal case. Conversely, links with the lower value of interaction become dominant in the cancerous network. It should also be pointed out that the two orange triangles between two dashed-lines of $PD_{0}$ plot (and equivalently the two orange long bars in $PB_{0}$) account for two small persistent clusters in the cancerous network. $\beta _{0}$-curve and correspondingly the number of arrows in $PB_{0}$ plot, confirms that both networks are path-connected for high weights. We further tested the contribution of gene interaction patterns to cellular networks by comparing the number of 1-dimensional holes (loops) in both networks, in which the graph of cancerous and healthy samples appeared to have deviated significantly. Figure 5 demonstrates the number of loops as a function of threshold. $PD_{1}$ and $PB_{1}$ plot illustrate that the cancerous network contains more persistent loops (persistence pairs between dashed-lines in $PD_{1}$ and long bars in $PB_{1}$) rather than the normal one. In the bottom panel of Fig. 5, $\beta _{1}$-curve reveals that the networks have reached the loopful regime at a distinct value of thresholds. According to the $PB_{1}$ plot and $\beta _{1}$-curve, there are several survived loops (arrows in $PB_{1}$ and tail of the curves in $\beta _{1}$-curve) in the cancerous network, while the normal network is almost loopless at the higher thresholds. We noticed that by increasing the weight of the interactions to its highest value, the number of loops in cancer samples does not reach zero. Our results suggest that studying the pattern of survived 1-dimensional holes can lead to the role of these persistent topological spaces in cancer networks.

**Figure 6:**
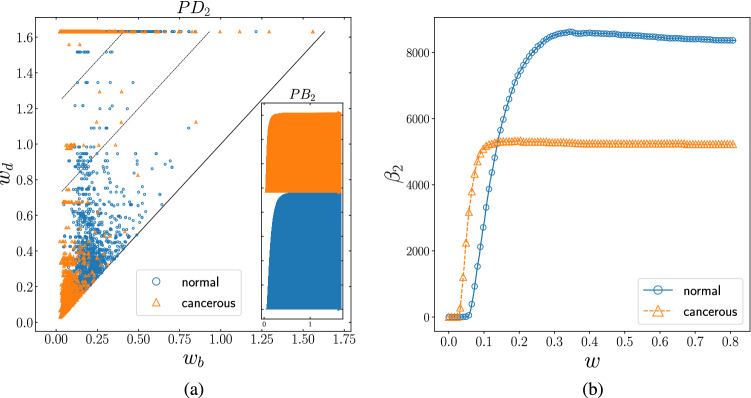
**(a)** Persistence diagram of 2-homology group ($PD_{2}$) for normal (blue circles) and cancerous (orange triangles) interaction network. The normal network contains more persistent voids (persistent pairs between dashed-lines) rather than the cancerous one. Inset: Corresponding persistence barcode ($PB_{2}$) for normal (blue bars) and cancerous (orange bars) network. The long bars correspond to the persistent voids, as well, the normal network has more survived void (arrows) rather than the cancerous network. **(b)** $\beta _{2}$-curve for normal (blue circle) and cancerous (orange triangle) network. The curves illustrate that the statistics of voids of the networks saturate in various value of threshold, such that the $\beta _{2}$-curve for the cancerous network saturates earlier (approximately 0.1) than the normal (approximately 0.5) network, while it saturates to the lower value (approximately 5000) than the normal case (approximately 8000).

Figure 6 compares the number of two-dimensional holes (voids) in these networks. The existence of the persistence pairs between dashed-lines in $PD_{2}$ along with the long bars of $PB_{2}$ suggests that the normal network includes more persistent voids compared to the cancerous one. As it can be remarked from this figure, the number of two-dimensional holes for both networks starts increasing at small values of threshold. The separation of the $\beta _{2}$-curves, however, illustrates that the statistics of the voids saturation is distinct in these two data sets, such that the $\beta _{2}$-curve for the cancerous network saturates at the smaller values of interaction.This separation of the pattern is evident at the higher value of the threshold where the last stage of filtration shows a prominent deviation in the number voids in these two states. According to the number of arrows in $PB_{2}$ plot and the tail of $\beta _{2}$-curve, one expects that the number of voids in the normal network is significantly higher. We conclude that unlike patterns of loops, voids are more dominant in the normal network in the high threshold region. Our weight distribution function analysis implies that the cancerous network includes a total number of links with weaker interactions compared to the normal case. The sharper weight distribution function of the cancerous network around smaller absolute values, reveals how this network goes through its topological evolution more promptly as their weights are more restricted to smaller values. The number of connected components dropped, the number of loops and voids raised, and the saturation all happened at smaller thresholds compared to the normal case. One biological interpretation of this result could be that genes in the cancerous cell seem to be highly dependent on specific pathways causing them to start interacting at smaller thresholds and finding their isolated pathways at smaller values. In this study, according to our results, we propose TDA can be employed to associate cancer cell proliferation to numbers and the evolution of topological features, so as to study this disease from the viewpoint of patterns of genes’ interaction in order to confirm how local topological modifications may contribute to global features and propose examining the patterns of interactions as a general and global picture as an alternative to studying single genes and their pairwise interactions.

## Conclusion

Adopting a novel computational approach, we propose that topological data analysis methods, such as Persistent Homology can be used to study cancer sample data to gain a better perspective on the complexity of this disease at the network level. Cancer is the most common human genetic disease, generated by a number of certain modifications into genes that control the way our cells function. Genes interact with each other, which their highly correlated expressions, and their interactions within a regulatory frame and leading to the emergence of complex structures in the cells, led researchers to investigate the Gene Regulatory Network (GRN) of cells in the framework of graph theory. In this study, we found that network structures are distinctive for normal and cancer samples in both the number and persistence of topological features. Biologically, it is possible that patterns of Betti curves in cancer samples are a manifestation of oncogene addiction at the network level. This phenomenon is defined based on experimental observations that cancer cells appear to be highly dependent on a specific oncogenic pathway^69^. It is plausible that the persistent topological spaces in cancer samples are sets of tightly related genes that modulate a specific oncogenic pathway, critical for cellular survival and proliferation. Referring back to our building analogy, with its floors and considering its building plan, our question now is if there exist some established patterns for the genes in cancerous networks upon which genes interact, or how these patterns, deviating significantly from the healthy one develop within the networks.
